# Low skeletal muscle mass and liver fibrosis in children with cerebral palsy

**DOI:** 10.1007/s00431-023-05177-9

**Published:** 2023-09-01

**Authors:** Marie Mostue Naume, Marianne Hørby Jørgensen, Christina Engel Høi-Hansen, Maja Risager Nielsen, Alfred Peter Born, John Vissing, Lise Borgwardt, Dorte Marianne Rohde Stærk, Mette Cathrine Ørngreen

**Affiliations:** 1 Copenhagen Neuromuscular Center, Department of Neurology, Copenhagen University Hospital, Rigshospitalet, Copenhagen, Denmark; 2 Department of Paediatrics and Adolescent Medicine, Copenhagen University Hospital, Rigshospitalet, Copenhagen, Denmark; 3 Department of Clinical Medicine, University of Copenhagen, Rigshospitalet, Copenhagen, Denmark; 4 Department of Clinical Physiology, Nuclear Medicine & PET, Copenhagen University Hospital, Rigshospitalet, Copenhagen, Denmark; 5 Department of Radiology, Copenhagen University Hospital, Rigshospitalet, Copenhagen, Denmark

**Keywords:** Cerebral palsy, Liver fibrosis, Steatosis, Low skeletal muscle mass, Altered body composition

## Abstract

The purpose of the study was to conduct a nutritional and metabolic assessment of children with cerebral palsy, including an investigation of liver status, body composition, and bone mineral density. In this cross-sectional study we included 22 children with cerebral palsy. By using ultrasound, transient elastography, dual x-ray absorptiometry (DXA) scan, blood samples, anthropometric measurements, and a three-day diet registration, the nutritional and metabolic status was evaluated. Liver fibrosis and steatosis were found in four patients (18.2%), all with severe motor impairments, low skeletal muscle mass, and epilepsy. All patients with liver involvement had normal liver-related blood samples. Decreased bone mineral density was found in 26.3%, and 91.0% had low skeletal muscle mass. Fat mass and muscle mass were significantly lower in the patients with severe motor impairments compared to the patients with less severe motor impairments. Within the children classified as ‘underweight’ or ‘normal’ according to body mass index, body fat determined by DXA scan was normal or high in 50% of these patients.

*Conclusions*: This study is the first to report liver fibrosis and steatosis in children with cerebral palsy. Possible causes of liver fibrosis and/or steatosis are altered body composition with low skeletal muscle mass, decreased mobility and medical drug intake. Further investigations of liver involvement and risk factors are needed.

**Table:** 

**What is Known:** *• Children and adolescents with cerebral palsy are at risk of malnutrition and altered body composition, both of which can lead to fatty liver disease*. *• It is unknown whether children with cerebral palsy are at increased risk of metabolic disturbances such as fatty liver disease*.	
**What is New:** *• Altered body composition and low skeletal muscle mass, regardless of ambulation is present in 91% of the children with cerebral palsy*. *• Liver fibrosis and/or steatosis were found in 18.2% of the patients. Possible causes are altered body composition, decreased mobility and medical drug intake*.

**What is Known:**

*• Children and adolescents with cerebral palsy are at risk of malnutrition and altered body composition, both of which can lead to fatty liver disease*.

*• It is unknown whether children with cerebral palsy are at increased risk of metabolic disturbances such as fatty liver disease*.

**What is New:**

*• Altered body composition and low skeletal muscle mass, regardless of ambulation is present in 91% of the children with cerebral palsy*.

*• Liver fibrosis and/or steatosis were found in 18.2% of the patients. Possible causes are altered body composition, decreased mobility and medical drug intake*.

## Introduction

Cerebral palsy (CP) is a group of chronic disorders of movement and motor function, caused by non-progressive injuries or abnormalities in the immature or developing brain [1]. Risk factors for CP include premature birth, neonatal asphyxia, injury caused by hypoxic-ischemic insults, infection, or genetic disease [1]. CP is the most common cause of motor dysfunction in children, affecting two out of 1000 newborns. Children with CP are at risk of nutritional disturbances, such as malnutrition, swallowing difficulties, and vitamin deficiencies [2–5]. The prevalence of low bone mineral density (BMD) is high in the patient group, especially in wheelchair-dependent children due to immobilization, malnutrition, feeding difficulties, nutrient deficiencies, and poor growth [2, 6, 7]. Many children with CP, in particular those with more severe motor impairments, have low muscle mass due to atypical muscle tone and immobilization [8–10]. Low muscle mass is found to be a risk factor for metabolic disturbances and non-alcoholic fatty liver disease (NAFLD) [11, 12]. NAFLD is the liver component of metabolic syndrome and is characterized by fat accumulation in the liver, which may develop further into liver fibrosis [13]. Obesity is one of the leading causes of developing NAFLD, however severe malnutrition in young children is also associated with signs of hepatic dysfunction such as steatosis [14]. Malnutrition is well-described in the patient group with an overall prevalence of 40% [15]. Malnutrition has a negative impact on quality of life and morbidity of the children. The optimal nutritional strategy is challenging, and reliable measurements of growth and body composition are difficult to obtain, due to fixed joint contractures, scoliosis, spasms, and cognitive defects [16]. Body mass index (BMI) and weight are used to assess growth and nutritional status, even though these measurements may be inaccurate due to the patients having altered body composition compared to typically developed children [17]. Whole-body dual-energy X-ray absorptiometry scans (DXA scan) are found to be the gold standard for measuring body composition in the patient group [18].

Furthermore, low birth weights and high birth weights are found to be risk factors for NAFLD in children [19]. Many patients with CP were born prematurely with low birth weight. It is unknown whether children with CP are at increased risk of metabolic disturbances such as NAFLD.

The study aimed to conduct a metabolic and nutritional assessment of children and adolescents with CP, including an investigation of the liver, body composition, bone mineral density, and macronutrients.

## Materials and methods

### Study design and participants

This cross-sectional study was part of a metabolic and nutritional screening of patients with motor dysfunction and disability connected to the Neuropaediatric outpatient clinic, Copenhagen University Hospital, Rigshospitalet. The study design and methods have been described in detail elsewhere [20]. The methods are summarised here. All children with CP (age 0–18 years) across all functional ability levels were invited to participate when they were seen in the outpatient clinic between December 2018 and November 2021. Exclusion criteria were children with other chronic diseases, age > 18 years or if participation in the study was judged not suitable for the patient due to clinical or social non-compliance. The gross motor function of the children was categorized by experienced neuropaediatricians into five different levels using the gross motor function classification system (GMFCS) [21]. Consistent with other studies, we divided the children into two groups; GMFCS I-II, defined as children with less severe motor impairments, and GMFCS III-V, defined as children with more severe motor impairments [22, 23]. Furthermore, the CP subtypes were recorded as spastic hemiplegia, diplegia or tetraplegia, dyskinetic or mixed phenotype. The participants and their parents were informed about the study orally and in writing and provided written consent before study-specific procedures.

### Study visit

The project consisted of one study visit including a DXA scan, ultrasound of abdominal organs, FibroScan, and blood samples. Demographic data were obtained from the medical journal by the project coordinator. The demographic data included age, birth weight, aetiology of CP, subtype of CP, medications, history of liver disease, anthropometric measurements, and ambulatory status. Height and weight were obtained from the medial files based on the clinical visit in the outpatient clinic or reported by the parents. Birth weight was categorized into four different categories, very low birth weight (< 1500 g; VLBW), low birth weight (1500 to 2500 g; LBW), normal birth weight (2500 to 4000 g; NBW) and high birth weight (> 4000 g, HBW) [19]. Body weight, height, and BMI were converted into corresponding z-scores [24].

### Liver assessment

The liver status was assessed by ultrasound examination, transient elastography (FibroScan), and blood samples analysed for liver biochemistry. As described in a previous article, the participants underwent an ultrasound (GE Healthcare LOGIQ E9) of the abdominal organs by a specialist in paediatric radiology [20]. On ultrasound, liver steatosis was defined as liver parenchyma with a bright pattern (hyperechogenic), while liver fibrosis was defined as liver parenchyma with coarse pattern. The same operator (DMR-S) performed the ultrasound examinations on all participants. A FibroScan (FibroScan 502 (Echosens, Paris, France)) was also used to assess liver fibrosis. Details of the FibroScan method have been previously published [20]. A measured median liver stiffness above 7 kPa was defined as significant liver fibrosis [25, 26]. The same operator (MM-N) performed the FibroScan on all participants, except for one. The blood samples were analysed for alanine aminotransferase (ALT), lactate dehydrogenase (LDH), gamma glutamyl transferase (GGT), alkaline phosphatase, bilirubin, albumin, INR, thrombocytes and lipid profile (cholesterol, LDL-cholesterol, HDL-cholesterol).

### Body composition and bone mineral density

Body composition was investigated by BMI-z-scores, weight z-scores, and by DXA scan (Lunar Prodigy Pro, GE Healthcare, Madison, WI, USA). The WHO cut-offs for underweight and overweight were used to classify body weight [24]. The method of DXA scanning has been previously published [20]. The measurements of fat mass (kg) and fat free mass (kg) were obtained from the DXA scan and used to calculate fat mass index (FMI) and fat free mass index (FFMI), based on previously published equations [20]. FFMI was used as a measure of muscle mass. The FFMI and FMI were compared to reference values from a healthy population of children [27]. FMI ≥ 85th percentile was defined as “mild overweight” and FMI ≥ 95th percentile was defined as “overweight” [28, 29]. Furthermore, FMI ≥ 5th percentile and ≤ 85th percentile was defined as “normal” and FMI ≤ 5th percentile was defined as “underweight”. We defined low skeletal muscle mass as FFMI ≤ 10th percentile. For children aged 2 to 5 years, FFMI reference data were not available, low muscle mass was determined by the child’s dependence on a wheelchair. Bone mineral density (BMD) in the lumbar spine and total body were measured by the DXA scan. Low BMD was defined as previously described, with a BMD z-score < -1.9 [20].

### Vitamins, minerals, and 3-day food record

The blood samples were analysed for vitamins (25-OH vitamin D, vitamin E, vitamin A, vitamin B12) and minerals (zinc, magnesium). The participants and parents were asked to complete an internet-based three-day food record (MadLog Classic Aps, Kolding, Denmark), to assess the intake of macronutrients. The following information was abstracted from the food record: actual energy intake (kcal/day), macronutrient distribution with carbohydrate intake (%), fat intake (%), and protein intake (g/kg/d, %). The macronutrient distribution and daily protein intake were compared to guidelines for food intake in Nordic children [30].

### Statistical analysis

This was a descriptive study of children with CP who are followed in our clinic at Rigshospitalet. We aimed to include as many children as possible seen in the outpatient clinic within the time period. Therefore, a power calculation was not made. R version 4.2.0 with R studio version 2022.02.2 **(**R Foundation for Statistical Computing, Vienna, Austria**)** was used to perform the statistical analyses. Continuous data are presented as mean with standard deviation (SD). Levene’s Test for Equality of variances was used. Categorical data are presented as numbers with percentages (%). Welch t-test and independent two-sample t-test for continuous variables and Fisher's Exact test for categorical variables were used to compare the children classified with GMFCS levels I-II with children classified with GMFCS levels III-V. Two-sided p < 0.05 was considered statistically significant. Not all patients participated in all examinations due to practical issues for the family or the child could not cooperate. The numbers and percentages presented are calculated from the exact number of patients that participated in the examination.

### Approval and registration

The study was performed in line with the principles of the Declaration of Helsinki. The study was approved by the National Committee on Health Research Ethics (H-18011574) and the Data Protection Agency (VD-2018–130). The data that support the findings of this study are not openly available due to reasons of sensitivity and are available from the corresponding author upon reasonable request.

## Results

Approximately 55 children with CP were considered eligible for the study. We asked around 40 children who fulfilled inclusion with no exclusion criteria’s if they were interested in participating, of whom 22 accepted. Of these, seven patients (31.8%) had GMFCS I-II, and 15 patients (68.2%) had GMFCS III-V (Table 1). Twelve of the patients had spastic CP with the majority having tetraplegic affection (Table 1). The mean age of the patients was 9 years (range 4–16 years). The BMI z-scores and weight for age z-scores were significantly lower in the patients classified as GMFCS level III-V compared to the patients classified as GMFCS level I-II (Table 1).

**Table 1:** Demographics of the children with cerebral palsy included in the study

**Demographics**	**All** **N = 22**	**GMFCS I-II** **N = 7**	**GMFCS III-V** **N = 15**	***p***^**a**^
**Gender (F/M)**	9 / 13	3 / 4	6 / 9	-
**Age (years)**	9 ± 3.6	7.4 ± 2.8	9.8 ± 3.9	0.087
**Subtype**				
Spastic				
Hemiplegia	4 (18.2%)	3 (13.6%)	1 (4.5%)	-
Diplegia	5 (22.7%)	4 (18.2%)	1 (4.5%)	
Tetraplegia	3 (13.6%)	0	3 (13.6%)	
Dyskinetic	6 (27.3%)	0	6 (27.3%)	
Mixed phenotype (spastic/dyskinetic)	4 (18.2%)	0	4 (18.2%)	
**Birthweight (g)**	2287 ± 1103	1945 ± 1314	2433 ± 1020	0.441
VLBW (< 1499 g)	5 (25%)	2 (33.3%)	3 (21.4%)	-
LBW (1500–2499 g)	4 (20%)	2 (33.3%)	2 (14.2%)	
NBW (2500–4000 g)	10 (50%)	2 (33.3%)	8 (57.1%)	
HBW (> 4000 g)	1 (5%)	0	1 (7.1%)	
**BMI for age z-score**	-1.5 ± 2.9	0.25 ± 0.66	-2.4 ± 3.2	0.008^*^
**Weight for age z-score**	-2.2 ± 3.4	0.03 ± 0.9	-3.3 ± 3.7	0.004^*^
**Gastric tube**	9 (41.0%)	1 (14.3%)	8 (53.3%)	0.164
**Gastric tube + oral feeding**	3 (13.6%)	1 (14.3%)	2 (13.3%)	1.0
**Low skeletal muscle mass**	20 (91.0%)	5 (71.4%)	15 (100%)	0.090
**Epilepsy**	8 (36.4%)	1 (14.3%)	7 (46.6%)	0.190
**Anti-seizure medications**	8 (36.4%)	1 (14.3%)	7 (46.6%)	-
**Aetiology**				
Perinatal asphyxia	3 (13.5%)	1(14.3%)	2 (13.3%)	-
Prematurity	7 (31.2%)	2 (28.6%)	5 (33.3%)	-
Prematurity and group B streptococcus	1 (4.5%)	1 (14.3%)	0	-
Congenital CMV	1 (4.5%)	1 (14.3%)	0	-
Maternal infection	1 (4.5%)	0	1 (6.7%)	-
Intracranial haemorrhage	2 (9.1%)	1 (14.3%)	1 (6.7%)	-
Intrauterine damage on thalamus	1 (4.5%)		1 (6.7%)	-
Prenatal periventricular damage	1 (4.5%)	1 (14.3%)	0	-
Genetic (22q11 deletion and Aicardi-Goutières syndrome)	2 (9.1%)	0	2 (13.3%)	-
Unknown aetiology	3 (13.5%%)	0	3 (20.0%)	-

The values are given as mean ± standard deviations or count with percentages

*F* female, *M* male, *g* gram, *VLBW* very low birth weight, *LBW* low birth weight, *NBW* normal birth weight, *HBW* high birth weight, *BMI* body mass index, *GMFCS* gross motor function classification system, *CMV* cytomegalovirus

^*^p-value < 0.05

^a^GMFCS level I-II versus III-V. P-values are calculated with independent two sample t-test and fisher’s exact test

### Liver assessment

**Fig. 1:**
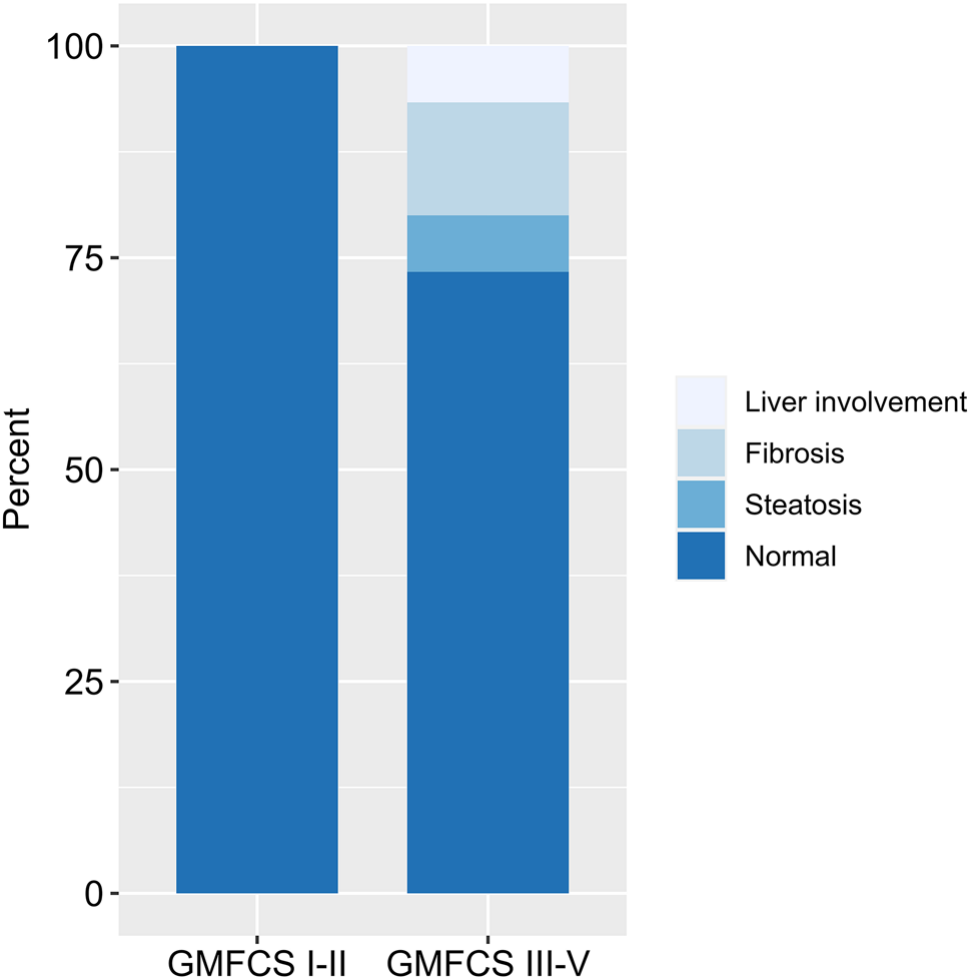
Percentage of liver fibrosis and/or steatosis assessed by ultrasound and FibroScan in children with cerebral palsy, grouped according to gross motor function. Abbreviations: Liver involvement = steatosis and fibrosis, GMFCS = gross motor function classification system

Liver fibrosis and/or steatosis was observed on FibroScan and/or ultrasound in 18.2% of the patients (n = 4). Fibrosis by FibroScan with liver stiffness ≥ 7 kPa was found in three patients; one of these had liver fibrosis on both ultrasound and by FibroScan. One patient had both steatosis by ultrasound and fibrosis by FibroScan. Lastly, one patient had steatosis by ultrasound. All patients with liver fibrosis and/or steatosis were categorized as GMFCS III-V (Fig. 1) and all had low skeletal muscle mass. Furthermore, two of the patients had dyskinetic CP and the two others had either spastic or a mixed phenotype. Three of the four patients were severely underweight or underweight according to BMI z-scores, whereas two of these had liver steatosis by ultrasound. All received daily medication due to epilepsy or movement disorder. Three were treated with daily anti-seizure medications, one patient was treated with diazepam (for epilepsy and dyskinesia), and two patients with levetiracetam. The fourth was treated with cannabinoid oil for dyskinesia. The ALT, LDH, bilirubin, thrombocytes, alkaline phosphatase, and lipid profile were normal in all patients with liver fibrosis and/or steatosis. None of the patients had a history of liver disease. Out of the total study population, 15 patients were assessed for liver fibrosis by FibroScan. The average median liver stiffness measured by FibroScan was normal in the total study population, with 5.0 kPa ± 1.64. The liver stiffness was not significantly different between the two groups of GMFCSI-II and III-V (median liver stiffness 4.2 kPa ± 0.7 vs. 5.4 kPa ± 1.9, p = 0.08). No association was found between birth weight and liver affection.

### Body composition and bone health

Severe underweight and underweight according to BMI-z scores were found in respectively two patients (9.1%) and four patients (18.1%). All underweight patients were GMFCS III-V. Overweight and obesity were found in two patients (9.1%), one with GMFCS I-II and one with GMFCS III-V. However, mild overweight and overweight measured by DXA scan were found respectively in five patients (29.4%) and one patient (5.8%). Table 2 shows that within the children categorized as severe underweight or underweight according to BMI z-score, four of the children had normal fat status measured by DXA. Furthermore, five patients with normal weight status according to BMI z-scores, were categorized as mild overweight or overweight by DXA. Out of the total study population, 91.0% had low skeletal muscle mass. The patients with GMFCS III-V had significantly lower FMI and FFMI compared to the patients with GMFCS I-II (Table 3).

**Table 2:** Classification of nutritional status according to BMI z-score and the corresponding classification of fat mass index by DXA scan

		**DXA scan (n = 17)**			
**Nutritional status**	**Total** **(n = 22)**	**Underweight**	**Normal**	**Mild overweight**	**Overweight**
**Severe underweight**	2 (9.0%)	1 (50.0%)	1 (50.0%)	0	0
**Underweight**	4 (18.2%)	0	3 (75.0%)	0	0
**Normal**	14 (63.6%)	0	6 (42.9%)	4 (28.6%)	1 (7.1%)
**Overweight**	1 (4.5%)	0	0	1 (100%)	0
**Obese**	1 (4.5%)	NA			

The number differentiates between BMI z-scores and DXA scan, due to some patients not being DXA scanned, or if the patients age were < 6 years, there were no FMI-percentiles available

*NA* not available, *BMI* body mass index, *DXA* dual energy x-ray absorptiometry scan

**Table 3:** Body composition and bone mass measured by DXA scan

**DXA Scan**	**All** **N = 19**	**GMFCS I-II** **N = 7**	**GMFCS III-V** **N = 12**	***p***^**a**^
Fat mass (kg)	7.3 ± 4.1	7.9 ± 4.4	6.9 ± 4.1	0.627
Fat mass (%)	28.3 ± 5.8	29.7 ± 7.4	27.4 ± 4.9	0.473
FMI	3.6 ± 2.0	4.9 ± 1.7	3.1 ± 1.9	0.045^*^
Muscle mass (kg)	17.6 ± 7.2	18.1 ± 6.9	17.3 ± 7.6	0.817
FFMI	9.04 ± 3.9	11.2 ± 0.9	8.0 ± 4.4	0.017^*^
BMD total	-0.5 ± 1.1	0.6 ± 1.1	-1.2 ± 0.5	0.009^*^
BMD lumbar spine	-1.02 ± 1.7	-0.53 ± 1.53	-1.34 ± 1.8	0.367
Low BMD total (%)	1 (5.3%)	0	1 (8.3%)	1.0
Low BMD lumbar spine (%)	4 (21.1%)	1 (14.3%)	3 (25.0%)	0.604

Values are given as mean and standard deviations or count with percentages

*DXA* dual energy x-ray absorptiometry, *kg* kilograms, *FMI* fat mass index, *FFMI* fat-free mass index, *BMD* bone mineral density, *GMFCS* gross motor function classification score

^*^p-value < 0.05

^a^GMFCS level I-II versus III-V. P-values are calculated with Welch two sample t-test or independent t-test

A total of five patients (26.3%) had low BMD, either in total body or in the lumbar spine (Table 3). One of these had vitamin D deficiency. The total BMD was significantly lower in the patients with GMFCS III-V compared to the group with GMFSC I-II. There was no association between vitamin D levels, calcium levels, or phosphate levels and low BMD. No one had experienced a fracture within the last 12 months.

### Vitamins, minerals, and 3-day food record

Vitamin D insufficiency (25-OH vitamin D < 50 nmol/l) and deficiency (25-OH vitamin < 25 nmol/l) were found in three patients (13.6%) and two patients (9.0%), respectively. Two of the patients with vitamin D insufficiency were classified as GMFCS-I and one were classified as GMFCS-V. The patients with vitamin D deficiency were classified as GMFCS-III and V, respectively. Four out of the five patients did not use vitamin D supplements. Vitamin E was slightly elevated or elevated in 13 patients (above normal range 7–21 µmol/L up to 12 years, and 14–23 µmol/L from 13–17 years). Furthermore, 13 patients (61.9%) had low levels of creatinine, of whom 11 were non-ambulant. Six patients had low levels of zinc. The triglycerides were normal in 20/22 patients. Nine patients (41.0%) used daily vitamin D supplements. A 3-day food record was completed in 31.8% (n = 7). The mean macronutrient distribution for this group was 46.7% ± 10.3 carbohydrates, 32.9% ± 8.4 fat, and 15.6% ± 2.9 proteins, all within the recommended guidelines for food intake in children [30]. The daily protein intake per kilogram body weight was above the recommended levels (0.8–1.5 g/kg/day) in the group, with an average of 2.8 g/kg/day ± 1.4.

## Discussion

In this cross-sectional study investigating the nutritional and metabolic status in a Danish group of children and adolescents with CP of whom 91% had low skeletal muscle mass, we found: a) 18.2% had liver fibrosis and/or steatosis, b) altered body composition and decreased BMD were prevalent, c) there was a discrepancy between assessing nutritional status by BMI z-score compared to fat status by DXA scan, d) nutrient deficiencies were prevalent in the study population. The nutritional and metabolic disturbances were seen mostly in the patients with severe motor impairment, with GMFCS level III-V.

This study is the first to describe liver fibrosis and steatosis in children and adolescents with CP. The overall prevalence of NAFLD in European children and adolescents is 2.7% [31]. The relatively high incidence of liver involvement found in our study (18.2%) may be explained by the patient’s altered body composition. Three out of the four patients were categorized as severe underweight or underweight according to BMI z-scores, indicating malnutrition. Malnutrition may increase the risk of liver steatosis and fibrosis. Although, two of the patients with liver involvement had normal fat status measured by DXA scan. As indicated in our recent study investigating hepatic manifestations in children affected by neuromuscular diseases, low skeletal muscle mass may increase the patient’s risk of developing liver involvement [20]. In the current evaluation of children with CP, all patients with liver involvement had low skeletal muscle mass and were severely affected by their disease with GMFCS III-V. Therefore, low skeletal muscle mass and decreased mobility may increase the risk of liver steatosis and fibrosis. Furthermore, glucose is disposed primarily in the skeletal muscle in an insulin-responsive manner, and the loss of muscle mass may lead to insulin resistance. Insulin resistance may enhance fat accumulation in the liver by increasing free fatty acid delivery [32]. Insulin resistance has been reported in overweight CP patients and in patients with spinal muscular atrophy, a neuromuscular disorder also characterized by low skeletal muscle mass [33, 34]. A larger focus on clinical care and future studies is warranted to investigate liver involvement in children with CP, as this is the first study to our knowledge describing this issue. Furthermore, all patients with liver involvement used daily medications for epilepsy or movement disorders. The medications are not commonly known for being potentially hepatotoxic, however, our results may indicate the need for investigating possible hepatic effects of anti-seizure medications in children and adolescents with CP in a larger study.

Nutritional status was assessed by two methods. In the patients categorized by BMI as severe underweight, underweight, or normal, the fat mass index measured by DXA scan revealed that many of the patients had a normal amount of fat or excessive fat status. In line with this finding, other studies have found BMI to be a poor predictor of body composition in the patient group [18, 22]. The anthropometric measurements and the calculated z-scores are used as standard assessments of growth in the clinic and for optimization of the nutrition. By using these measurements without other measurements of body composition such as DXA scan or triceps skinfold thickness [17], there may be a risk of overfeeding, especially in patients with gastrostomy tubes [35]. This may result in an excess amount of body fat increasing the risk of cardiovascular disease and metabolic syndrome. This has been documented in a study by Heyn and co-workers, who described a high prevalence of metabolic syndrome in adult patients with CP [36]. CP-specific growth charts are available, and using these may lower the risk of overfeeding. The CP charts demonstrate significant deviations from the general population reference centiles [37–39]. However, using these growth charts is under discussion. Center for disease control (CDC) and the European Society of Paediatric Gastroenterology, Hepatology and Nutrition (ESPGHAN) do not recommend the disease-specific growth charts due to limitations such as small sample size, data is not from a well-nourished group of children, and failure to consider secondary medical conditions. Nonetheless, there is a growing interest in the use of the charts and the possibility of recognizing limited growth potential to avoid invasive feeding approaches that are not needed.

In line with other studies, we found a high percentage of patients with vitamin and mineral deficiencies [40–43]. Vitamin D deficiency and insufficiency is well-described in the patient group, with a negative impact on bone formation and bone mineral density [40]. The poor exposure of sunlight, poor nutritional intake and immobility in the patient group increase the risk of vitamin D deficiency. Thus, it is recommended that the patient group uses daily supplements of vitamin D. Most of the patients with vitamin D deficiency or insufficiency in our study population did not use vitamin D supplements, despite having received information on this from treating clinician, explaining our finding. Poor nutritional intake and malnutrition may also explain the high percentage of patients with low levels of zinc. Other studies have found a high prevalence of children with CP with low levels of zinc, also in relation to low intake of zinc in the diet [35, 42, 43]. Although clinicians recommend that the patients take daily multivitamin supplements, it would be beneficial with regular measurements of vitamins and micronutrients as part of standard follow-up. Lastly, the high percentage of patients with low creatinine is probably explained by the patient’s low skeletal muscle mass. It is well established that children with CP can have multiple associated and secondary medical conditions and that management requires a multidisciplinary approach. In the NICE guideline [44] bone health has a specific focus and it is recommended to measure DXA and bone-related blood samples if the patient has one or more risk factors for low bone mineral health. Our results indicate that children may have reduced bone health also if they do not have a risk factor for this. Furthermore, there is also a point in the NICE guideline regarding diet, but there is not a section regarding liver status or awareness regarding the unfortunate combination of low muscle mass and possible hepatic affection of drug intake. We recommend an increased focus on liver status, especially in children who have low skeletal muscle mass.

This study was strengthened by the systematic evaluation of the liver and the body composition, but limited by the small number of participants, as well as the cross-sectional design being unable to confirm a cause-effect relationship between risk factors and liver fibrosis and steatosis. Furthermore, it is possible that the discrepancy between the nutritional status assessed by anthropometry and DXA scan is due to the weight and height were recorded from the medial files. Future studies investigating liver disease in CP patients should transform measurements of coarse (fibrosis) and bright (steatosis) patterns by ultrasound to indexes of steatosis and fibrosis to quantify the involvement or use other modalities, as seen in a study of patients with the neuromuscular disorder Duchenne muscular dystrophy [45].

## Conclusion

This study is the first to report liver fibrosis and steatosis in children with CP. Possible causes of liver fibrosis and steatosis are altered body composition with low skeletal muscle mass and decreased mobility. Further investigations of possible liver fibrosis and steatosis in the patient group are needed.

## Abbreviations

ALT: Alanine amino transferase
BMD: Bone mineral density
BMI: Body mass index
CP: Cerebral palsy
DXA: Dual-energy X-ray absorptiometry scan
FMI: Fat mass index
FFMI: Fat free mass index
GGT: Gamma glutamyl transferase
GMFCS: Gross motor function classification system
HDL: High density lipoprotein
HBW: High birth weight
LBW: Low birth weight
LDH: Lactate dehydrogenase
LDL: Low density lipoprotein
NAFLD: Non-alcoholic fatty liver disease
NBW: Normal birth weight
VLBW: Very low birth weight
